# Apolipoprotein A-I, A-II, and H mRNA and protein accumulation sites in the developing lung in late gestation

**DOI:** 10.1186/1756-0500-4-235

**Published:** 2011-07-14

**Authors:** Mélissa Côté, Pierre R Provost, Yves Tremblay

**Affiliations:** 1 Reproduction Axis, Perinatal and Child Health, Rm T-1-49, CHUQ Research Center, Québec City, Québec, Canada; 2 Department of Obstetrics and Gynecology, Faculty of Medicine, Laval University, Québec City, Québec, Canada; 3 Centre de Recherche en Biologie de la Reproduction (CRBR), Laval University, Québec City, Québec, Canada

## Abstract

**Background:**

Expression of apolipoprotein A-I (apoA-I), A-II, and H was previously observed at 16 to 50-fold higher levels in the fetal than the adult mouse lung. Here, sites of apoA-I, A-II, and H mRNA and protein accumulation were determined in mouse fetal lungs by *in situ *hybridization and immunohistochemistry in late gestation.

**Results:**

Expression sites vary for the three genes and change for the distal epithelium before the end of the canalicular stage, thus where and when the surge of surfactant synthesis occurs. Messenger of apoH, but not those of apoA-I and A-II, was also observed in the proximal epithelium and smooth muscles surrounding arteries. In contrast to apoC-II protein, none of the three studied apolipoproteins accumulated within secretory granule-like structures. Immunohistochemistry revealed that apoA-I and apoH accumulated mainly in capillaries. Three different positive signals with the anti-apoA-II antibody were found: one transient signal in the nucleus of a portion of mesenchymal cells, a second at lower levels throughout the mesenchyme, and another in capillaries with a specific increase from gestation day 17.5/18.5.

**Conclusion:**

Temporal and geographic co-expression of apoAI, AII, and H genes with surfactant production site suggests that the three apolipoproteins are secreted to play roles supporting the lung-specific surfactant lipid-related metabolism.

## Background

It is well recognized that the incidence and the severity of respiratory distress syndrome (RDS) affecting preterm neonates presents a sex difference with a disadvantage for males [1-5]. This sex difference was attributed to the effect of androgens in males which delay the surge of surfactant synthesis [2,6-10]. Recently, we reported in a real time quantitative PCR (QPCR) study that four apolipoproteins, namely, apolipoprotein A-I (apoA-I), apoA-II, apoC-II, and apoH, are expressed in the fetal mouse lung with a sex difference (P = 0.0896, 0.0896, 0.0195, and 0.0607 respectively) [11]. In addition, an increase in apoA-I-, apoA-II-, apoC-II-, and apoH mRNA levels was observed from gestation day (GD) 16.5 to GD 17.5 in correlation with the emergence of mature type II pneumonocytes [11]. Accordingly, lipoprotein lipase (LPL) mRNA was found in the developing lung with stable levels over time from GD 15.5 to 17.5, followed by a statistically significant small increase from GD 17.5 to 18.5.

Surfactant synthesis necessitates fatty acids, which can be provided by de novo synthesis or triglyceride-rich lipoproteins through LPL activity. When activated by its essential co-factor, apoC-II, LPL catalyzes cleavage of acyl-glycerol esters in triglycerides of circulating VLDL and chylomicrons. A role for LPL in surfactant synthesis was proposed [11-14]. In many tissues including adipose tissue and skeletal muscle, delivery of fatty acids from triglyceride-rich lipoproteins occurs by hydrolysis on the luminal surface of the capillary endothelium. This is also the major localization site for LPL protein in the developing lung [12]. Recently, we also showed that apoC-II and LPL mRNAs correlate temporally and geographically with surfactant lipid synthesis in preparation for birth [12] and that apoC-II is present in secretory granule-like structures located near the basal membrane of the distal epithelia [11] with no or small lumina during a short perinatal period [12]. Taken together, our results suggested that fatty acid recruitment from the circulation by apoC-II-activated LPL could be regionally controlled by modulation of apoC-II secretion [12] for the purpose of surfactant synthesis.

ApoH was reported to play a role in triglyceride removal from the plasma [15] and to enhance apoC-II-activated LPL activity [16]. ApoA-I and apoA-II are known to be involved in lipid transport [17,18] and a role for apoA-II in triglyceride metabolism was suggested (see review [18]). Therefore, a role for these apolipoproteins in fatty acid recruitment from triglycerides for surfactant lipid synthesis can be postulated. Because apoA-I, apoA-II, and apoH were co-regulated with apoC-II both over developmental time and from sample to sample in our previous QPCR study with whole lungs [11], it would be relevant to determine whether similar patterns of mRNA and protein accumulation sites, including the presence of apolipoproteins in secretory granules, are common features to all these apolipoproteins. In the present study, we determine similarities and differences between these apolipoproteins in their mRNA and protein distribution in the developing lungs over gestation time. Using *in situ *hybridization and immunohistochemistry, we show that despite several similarities, major differences exist between apolipoproteins. Time-dependent accumulation of the positive apoA-II epitope in association with the nucleus of several mesenchymal cells is a noteworthy novel observation.

## Results

It should be noted that all the results reported here were reproduced for two fetuses of three different litters for each time point.

### ApoA-I

**Figure 1:**
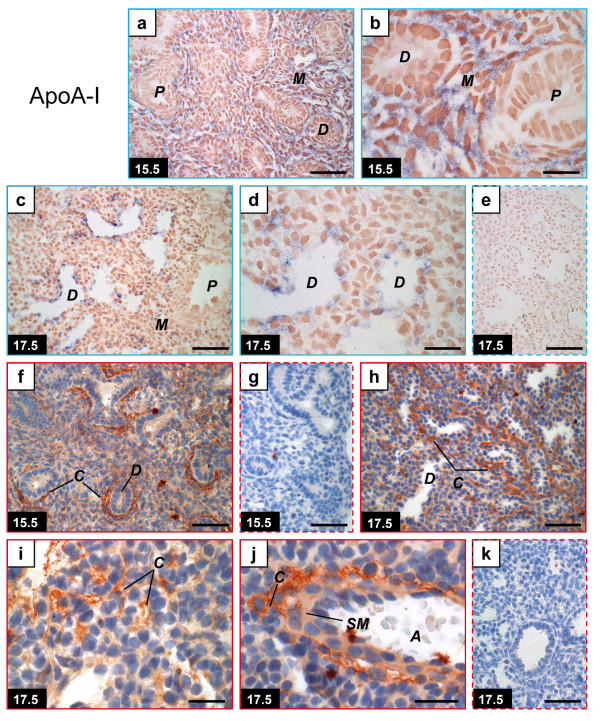
**Distribution of apolipoprotein A-I mRNA and protein in the mouse fetal lung**. Mouse tissue sections are from pseudoglandular (GD 15.5: a-b, f-g) or late canalicular (GD 17.5: c-e, h-k) stages. *In situ *hybridization (within blue frames) (a-e) was performed with apoA-I anti-sense (a-d) and sense (e) probes. Positive signals (blue) show that the site of apoA-I mRNA synthesis changes according to developmental time. Immunohistochemistry (within red frames) (f-k) was performed using an anti-apoA-I polyclonal antibody (f, h-j) or goat IgG as negative control (g, k). Positive signals (red) were mainly found on capillaries with no change in localization between GD 15.5 and 17.5. GDs are indicated on photographs. Dashed frames, negative controls. Scale bars, 50 μm (a, c, e-h, k) or 20 μm (b, d, i-j). ***A***, artery; ***C***, capillaries; ***D***, distal epithelium; ***M***, mesenchyme; ***P***, proximal epithelium; ***SM ***smooth muscle.

As demonstrated by *in situ *hybridization, the site of *apoA-I *gene expression changes between GD 15.5 and GD 17.5 (Figure 1a-e). On GD 15.5, mRNA was found almost exclusively in mesenchymal cells. In contrast, on GD 17.5, positive signals were found on epithelial cells of the distal epithelium, but not in the proximal epithelium and the mesenchyme. A week signal was observed in the mesenchyme on GD 16.5 (data not shown). These results were confirmed by using a second apoA-I RNA probe (data not shown).

The apoA-I protein was then localized by immunohistochemistry. In contrast to apoA-I mRNA, the apoA-I protein was found in similar structures from GD 15.5 to GD 17.5 (Figure 1f-k and data not shown). A strong positive signal was observed mainly in capillary-like structures, while a diffuse weak signal was observed throughout the tissue sections. An example of capillaries in fetal lungs is shown in our recent publication (platelet endothelial cell adhesion molecule-1 (PECAM-1)-positive structures in Figure three of [12]). No major change in sites of apoA-I accumulation was observed over developmental time, except a possible decrease in the intensity of the diffuse signal, but little variations from sample to sample prevent us from drawing a definitive conclusion.

### ApoA-II

**Figure 2:**
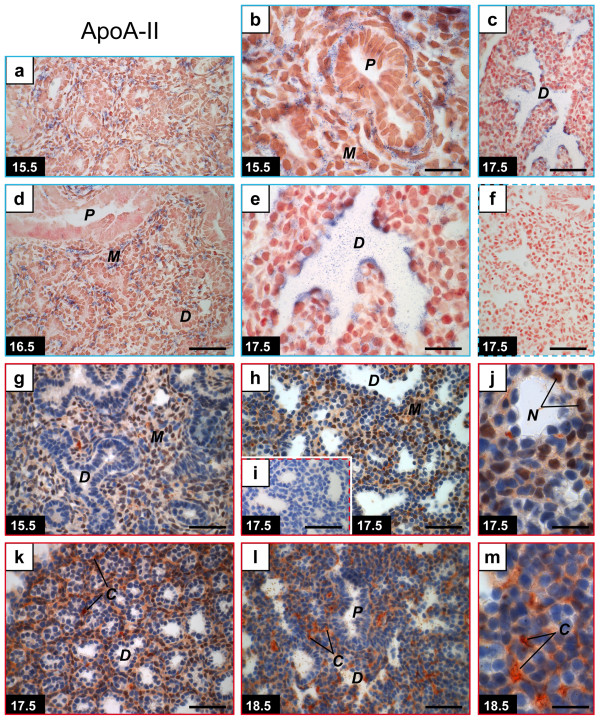
**Distribution of apolipoprotein A-II mRNA and protein in the mouse fetal lung**. Mouse tissue sections are from pseudoglandular (GD 15.5: a-b, g), junction between pseudoglandular and canalicular (GD 16.5: d), late canalicular (GD 17.5: c, e-f, h-k), or early saccular (GD 18.5: l-m) stages. *In situ *hybridization (within blue frames) (a-f) was performed with apoA-II anti-sense (a-e) and sense (f) probes. Positive signal is blue. A change in sites of apoA-II gene expression was observed according to gestation time. Immunohistochemistry (within red frames) (g-m) was performed using an anti-apoA-II polyclonal antibody (g-h, j-m) or goat IgG as negative control (i). Positive signal (red) was found in the mesenchyme. Some mesenchymal cells presented a stained nucleus between GD 15.5 and GD 17.5. Positive signals were also observed on capillaries. GDs are indicated on photographs. Dashed frames, negative controls. Scale bars, 50 μm (a, c-d, f-i, k-l) or 20 μm (b, e, j, m). ***C***, capillaries; ***D***, distal epithelium; ***M***, mesenchyme; ***N***, positive nuclei; ***P***, proximal epithelium.

Similarities were found between the apoA-I and the apoA-II gene expression patterns. As for apoA-I, the major site of apoA-II expression switches from the mesenchyme to the distal epithelium before the end of the canalicular stage (Figure 2a-f compared to Figure 1a-e for apoA-I). However, the positive signal observed for apoA-II by *in situ *hybridization on GD 15.5 and 16.5 (Figure 2a-b, d) is more cell specific than that of apoA-I (Figure 1a-b) in that it was mainly found in clusters of mesenchymal cells. As for apoA-I, the mesenchyme and the distal epithelium were respectively negative for apoA-II on GD 17.5 and GD 15.5, while the proximal epithelium was always negative. It should be noted that the structure corresponding to the most distal epithelium on GD 15.5 is different from that on GD 17.5, the latter being more differentiated.

**Figure 3:**
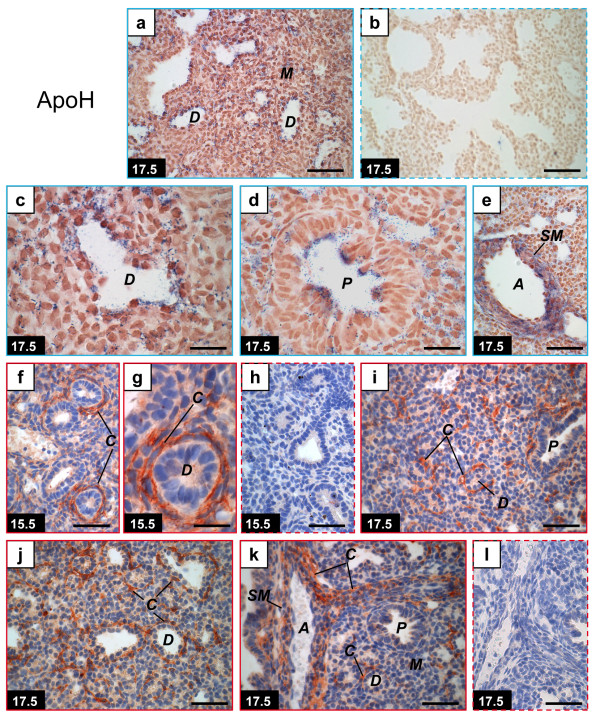
**Distribution of apolipoprotein H mRNA and protein in the mouse fetal lung**. Mouse tissue sections are from pseudoglandular (GD 15.5: f-h) or late canalicular (GD 17.5: a-e, i-l) stages. *In situ *hybridization (within blue frames) (a-e) was performed with apoH anti-sense (a, c-e) and sense (b) probes. Positive signals (blue) were found on GD 17.5 in the distal and proximal epithelia and smooth muscles surrounding large arteries. Immunohistochemistry (within red frames) (f-l) was performed using an anti-apoH polyclonal antibody (f-g, i-k) or goat IgG as negative control (h, l). Positive signals (red) were mainly found on capillaries with no change in localization between GD 15.5 and 17.5. GDs are indicated on photographs. Dashed frames, negative controls. Scale bars, 50 μm (a-b, e-f, h-l) or 20 μm (c-d, g). ***A***, artery; ***C***, capillaries; ***D***, distal epithelium; ***M***, mesenchyme; ***P***, proximal epithelium; ***SM ***smooth muscle.

Three types of positive signals were obtained by immunohistochemistry for apoA-II (Figure 2). The first one had a weak to medium intensity and spread throughout the mesenchyme; the second was found on the nucleus of several but not all mesenchymal cells; and the third was found on capillaries. Obviously, the diffused signal in the mesenchyme was not associated to apoA-II producing cells both on GD 17.7 when the gene is rather expressed in epithelial cells, and on GD 15.5 when the protein signal was not restricted to the clusters of mesenchymal apoA-II producing cells. Nuclei positive for apoA-II protein were observed on GD 15.5 and GD 17.5 (Figure 2g-k) but not on GD 18.5 (Figure 2l-m) and are thus a gestation time-dependent feature. The fact that apoA-II gene was not expressed in the mesenchyme on GD 17.5 strongly suggests that the nuclear anti-apoA-II-positive proteins were internalized by positive cells from the extracellular microenvironment. Such a nuclear signal was not observed for apoA-I (Figure 1), apoH (Figure 3) and apoC-II [11,12]. An obvious apoA-II positive signal on capillaries, similar to that obtained for apoA-I, was observed for one third of the tissues from GD17.5 (Figure 2k and data not shown) and all the samples from GD 18.5 (Figure 2l-m and data not shown). In contrast, a weaker positive signal was detected on capillaries for samples from GD 15.5 (Figure 2g and data not shown) and two third of the samples from GD 17.5 (Figure 2h and data not shown). Taken together, our results are compatible with an increase in apoA-II protein accumulation on capillaries over gestation time with significant levels from GD 17.5/18.5.

### ApoH

There are great similarities between apoH and LPL [12] localization of mRNAs and proteins. Both proteins were found in capillary-like structures on GD 15.5, GD 16.5, and GD 17.5 (Figure 3f-l, [12] and data not shown) and both mRNAs were found in epithelial cells of the distal epithelium on GD 17.5 (Figure 3a-c, [12]). In contrast to apoA-I and apoA-II, apoH was generally expressed in the proximal epithelium (Figure 3d). Some cells of the proximal epithelium were also positive for LPL [12]. The amounts of apoH mRNA on GD 15.5 (8 fetuses from 4 litters) and GD 16.5 (5 fetuses from 3 litters) were below the detection limit by *in situ *hybridization (data not shown), although apoH mRNA was detected by QPCR on these gestation times [11].

ApoH mRNA was also observed in smooth muscle surrounding large arteries (Figure 3e), while no hybridization signal was observed in this structure for apoA-I and apoA-II (data not shown). ApoH (Figure 3k) and LPL [12] proteins were found in smooth muscles of arteries, but signal intensities were lower than those found in adjacent capillaries. A similar result was obtained for apoA-I protein (Figure 1j).

## Discussion

For apoA-I, apoA-II and apoH, our data show that mRNAs and proteins do not accumulate at the same sites. This is expected for secreted proteins. Messenger RNA localization sites changed according to gestation time similarly for the three studied apolipoproteins and apoC-II [12] in that the mRNAs were present in the distal epithelium on GD 17.5 but not on GD 15.5. Knowing that the surge of surfactant synthesis occurs in the distal epithelium on GD 17.5 in the mouse [19-21], a role for these four apolipoproteins in association with surfactant synthesis in the developing lung is suspected on the basis of gene expression. In contrast, there are some differences in mRNA accumulation sites on GD 15.5. While apoA-I mRNA was found throughout the mesenchyme (Figure 1a-b), apoA-II mRNA was found only in clusters of mesenchymal cells (Figure 2a-b) whereas apoH mRNA was not found (data not shown), which could be attributed to the fact that apoH mRNA is less abundant than mRNAs encoding for the other analyzed apolipoproteins [11]. In the mouse, levels of mRNAs encoding for apoA-I, apoA-II, and apoH are very high in fetal lungs compared to adult lungs where only 2 to 6% of the fetal levels were found by QPCR, in contrast to apoC-II mRNA which showed similar levels for fetal and adult lungs [11]. A similar situation was found for human with higher pulmonary mRNA levels for apoA-I, apoA-II, and apoH between the 32-35 weeks' gestation period compared to adulthood, and similar apoC-II mRNA levels for these two periods [22]. Therefore, transient roles for apoA-I, apoA-II and apoH are expected in the developing lung.

The protein accumulation sites presented more differences between apolipoproteins than the mRNA accumulation sites. Firstly, none of the three studied apolipoproteins were found in secretory granules on GD 17.5, which is a major difference compared to apoC-II [11,12]. Therefore, the postulated control of apoC-II secretion according to growth of the distal epithelium [12] is not a common feature to all apolipoproteins secreted in the lung in late gestation. However, this does not exclude the possibility that one or some other apolipoproteins may participate in surfactant synthesis with apoC-II. Accordingly, apoH was shown to enhance apoC-II-activated LPL activity [16] and its presence in capillaries in fetal lungs is compatible with an effect on LPL activity. A role for apoA-II in triglyceride metabolism was also suggested [18].

The widely distributed low positive signals obtained with the anti-apoA-I and the anti-apoH antibodies and the general signal obtained for apoA-II in the mesenchyme could correspond to local lipid transport. Whether lung-originating apoA-I, apoA-II and apoH interact with several cells before reaching capillaries, where strong positive signals were found, is not determined but is a plausible hypothesis. We know that ATP-binding cassette transporter A-I (ABCA-I) promotes transfer of cholesterol and phospholipids from cells to lipid-free apolipoproteins, particularly apoA-I, initiating HDL formation [18,23]. In the lung, ABCA-I was found in macrophages [24] and in type I and type II pneumonocytes [25,26] while *Abca*^*-/- *^mice showed severe respiratory distress, lung congestion, and bronchopulmonary dysplasia [27].

Plasma phospholipid transfer protein (PLTP) was shown to bind both purified apoA-I and apoA-II [28] and the lung is one of its major sites of gene expression [29,30]. In addition to its roles in lipoprotein metabolism [31], PLTP was proposed to play an integral role in surfactant lipid trafficking and reutilization in type II pneumonocytes, where it was shown to be expressed [32]. PLTP expression was also reported during late gestation [33] when high apoA-I and apoA-II expression was found [11]. Whether binding of apoA-I and apoA-II to PLTP occurs in the developing lung and has a physiological relevance remains to be determined.

An increase in apoA-II expression was reported to inhibit hydrolysis of VLDL and chylomicron triglycerides by LPL [34]. This should be explained at least in part by the capability of apoA-II to displace apoC-II from lipoproteins [35]. Such an effect could be attributed in the fetal lung to the apoA-II positive signal present in lung capillaries and increasing with gestation time. Therefore, apoA-II could participate to the regulation of the amount of phospholipids entering in the developing lung.

In a proteomic study, apoA-I precursor and apoA-IV were found in lamellar bodies in adult rat lungs [36]. While higher apoA-I mRNA levels were observed in fetal lungs compared to mature lungs in mouse and human, no apoA-I signal was found by immunohistochemistry in association with granule structure in our study. It would be surprising that enough apoA-I protein be present in lamellar bodies for observation of granules by immunohistochemistry in light microscopy. This is different from apoC-II-containing secretory granules that were found near the basal membrane of the distal epithelia, close to the mesenchyme [11,12], which should not be secreted in the lumina but rather in the tissue to target capillary-anchored LPL.

ApoA-I was already reported to have anti-inflammatory effects [37-39]. It was decreased in subjects with idiopathic pulmonary fibrosis while intranasal apoA-I treatment in the mouse showed a protective effect against the development of experimental lung injury and fibrosis [40]. The study of *apoA-I *^*-/- *^mice revealed that apoA-I plays important roles in limiting lung inflammation and oxidative stress [41]. ApoH was reported to be part of a complex antigen inducing anti-phospholipid autoantibodies [42,43]. Other studies are requested to know whether these properties of apoA-I and apoH are exerted in the fetal lung.

Interestingly, immunohistochemistry positive signals for apoA-II were observed on the nucleus of several but not all mesenchymal cells until GD 17.5 but not on GD 18.5 (Figure 2). Counterstaining with Mayer's hematoxylin can explain the dark-red color of the nuclear positive signals. Nuclear localization was also reported in specific experimental conditions for other apolipoproteins such as apoA-I [44], apo E [44,45], apo D [46], apoJ [47], and apoB [44], while further investigations revealed that apoB immunoreactivity was rather perinuclear [48]. A tetrahydrocortisol-apoA-I complex was shown to increase gene expression and rate of protein biosynthesis in hepatocytes, and to interact specifically with DNA elements [49]. However, in the developing lung, no nuclear signal was observed for apoA-I, (Figure 1), apoH (Figure 3), and apoC-II [12]. Whether the apoA-II epitope in nuclei corresponds to gene regulation by apoA-II remains to be demonstrated, but our results demonstrate that this characteristic is cell-specific and time-specific.

Lung cell and explant cultures are not promising models to study the effect of apolipoproteins on lung development and metabolism. Indeed, functional studies of apolipoproteins expressed in the developing lung should have to be done in vivo because the role of these proteins most likely involves lipid exchange with circulating blood. Adding to the complexity of the study of apolipoproteins function(s) in the lung is the fact that circulating lipids are only one of the two possible sources of fatty acids for surfactant lipid synthesis. As discussed elsewhere [12], de novo synthesis through fatty acid synthase as the only source of fatty acids in animal models can support surfactant synthesis, as evidenced by the fact that LPL and apoC II (the co-factor of LPL) deficiencies are not associated with respiratory distress syndrome and with a lack of surfactant [50,51]. The importance of the study of apolipoproteins in the developing lung lies in the fact that preterm birth frequently leads to surfactant insufficiency and therefore, local lipid transport that must involve local production of apolipoproteins may become an interesting pharmaceutical target in that context. Similarly, the fact that apoA I knockout mice survive at birth without respiratory distress [52] does not mean that apoA I is not related to surfactant lipid metabolism. In contrast, several observations suggest the involvement of apoA-I, A-II, C-II and H in the lipid metabolism related to the surge of surfactant synthesis: apoA-I, A-II, C-II and H genes present a narrow peak of elevated expression in human fetal lungs during the 32-35 week gestation window in correlation with the reported decrease in the incidence and severity of respiratory distress syndrome (RDS) [22]; apoA-I, A-II, C-II and H mRNAs show an increase from GD 16.5 to 17.5 in the mouse in correlation with the emergence of mature type II pneumonocytes [11] and, as shown in this report, in correlation with a change in the site of apolipoproteins expression favoring the distal epithelium where the surge of surfactant synthesis occurs. Furthermore, it is reported that VLDL-triglyceride concentrations increased drastically in the cord blood of preterm neonates from 32-34 weeks' gestation and that most of the neonates with RDS in that study were born before the timing of the drastic VLDL-triglyceride increase [53,54]. Accordingly, maternal loading with VLDL stimulates surfactant synthesis in rats [14] while in a group of preterm infants weighing less than 2000 g, lower cord blood total fatty acids levels were found in RDS infants compared with non-RDS infants [55]. In conclusion, the fact that knockout of genes do not lead to death or respiratory distress in term pups does not eliminate the potential for these genes to be important for survival in cases of preterm birth. Therefore, lung-originating apoA-I, A-II, C-II and H may efficiently contribute to the survival of preterm infants. In vivo approaches are requested to demonstrate this hypothesis.

## Conclusion

Our data show that apoA-I, apoA-II and apoH mRNAs are regulated temporally according to their expression sites, with the distal epithelium as their major site of expression on GD 17.5 when the surge of surfactant synthesis occurs. The study of protein localization revealed major differences compared to apoC-II in that none of these three apolipoproteins were found in secretory granules. ApoA-I and apoH were mainly found in capillaries while the distribution of apoA-II was more complex, with three distinct positive signals: one of weak to medium intensity spread throughout the mesenchyme, a second in nuclei of one fraction of mesenchymal cells that disappeared before GD 18.5, and a third increasing in intensity over developmental time in capillaries. Temporal and geographic co-expression of apoAI, AII, and H genes with surfactant production site suggests that the three apolipoproteins are secreted to play roles supporting the lung-specific surfactant lipid-related metabolism.

## Methods

### Mouse tissue preparation

Protocols were approved by the Animal Care and Use Committee and the Institutional Review Board of the Centre de Recherche du Centre Hospitalier Universitaire de Québec (protocols no. 2005-091 and 2008-071-2). Female and male Balb/c mice (Charles River Laboratories St-Constant SA, St-Constant, Qc, Canada) were mated during the night (mating window ± 8 h). The day of copulatory plug was considered as GD 0.5 (term GD 19.5). Pregnant females were killed by exposure to a CO_2 _atmosphere. The fetal sex was identified by examination of the genital tract. Confirmation of individual sex was done by PCR amplification of the Sry gene. Fetal lungs were collected and either kept frozen until RNA extraction or fixed in 4% buffered paraformaldehyde for 48 h at 4°C. Tissues were paraffin-embedded and cut in 5 μm slices. *In situ *hybridization and immunohistochemistry were performed on samples from one female and one male of three litters for each gestation day studied.

The surge of surfactant synthesis occurs on gestation day (GD) 17.5 in the mouse as indicated by the appearance of lamellar bodies [19], an increase in surface activity in the mouse lung homogenate [19], and by increases in the activity of some enzymes involved in pulmonary lipid metabolism [20,21].

### RNA probes and *in situ *hybridization

Specific amplicons were synthesized from fetal lung cDNA using oligonucleotides designed to span at least one intron. Amplified gene/GenBank accession number/position of the amplified sequence/5' oligonucleotide/3' oligonucleotide (sequences include one restriction site for sub-cloning): *ApoA-I*, NM_009692/first probe/167-414/GGGGAATTC-TATGTGGATGCGGTCAAAGA/GGGAAGCTT-TAGGGCTGCACCTTCTGTTT/second probe/451-765/GGGGAATTC-GAGCTCTACCGCCAGAAGG/GGGAAGCTT-ATCAGACTATGGCGCAGGTC; *ApoA-II*/NM_013474/99-473/GGGGAATTC-CCATCTGTAGCCTGGAAGGA/GGGAAGCTT-CCTTCCGCATTTATTGGAGA; *ApoH*/NM_013475/129-430/GGGGAATTCC-GGTTGTCCCCTTAAAGACA/GGGAAGCTT-ATCTGGGCTCCATTTTCCTT. These amplicons were cloned into pGEM-4Z (Promega Corp., WI, USA). DNA matrix for SP6 and T7 polymerases were prepared by PCR amplification of each of the subcloned amplicon with the oligonucleotides GGATTTAGGTGACACTATAGAATA and TAATACGACTCACTATAGGGAGAC, which overlap the 5' end of the SP6 and the T7 promoters, respectively. Then, RNA probes were prepared using digoxigenin (DIG)-UTP substrate (Roche Diagnostics, Qc, Canada) and SP6 (sense) or T7 (antisense) RNA polymerases (Roche Diagnostics), as previously described [56]. *In situ *hybridization was performed as reported [56] except that denatured DIG-cRNA probes were used at 5 ng/μl. Slides were counterstained with 0.25% neutral red.

### Immunohistochemistry

Tissues were deparaffinized and subjected to immunohistochemistry as reported [56]. All the anti-apolipoprotein antibodies were purchased from Santa Cruz Biotechnology Inc. (CA, USA): goat anti-apoA-I (K-20) (1:20), goat anti-apoA-II (L-20) (1:20), goat anti-apoH (N-13) (1:20). A goat IgG preparation (Vector Laboratories Inc, ON, Canada) was used instead of primary antibody as negative control. A biotinylated donkey anti-goat IgG (Millipore Canada Ltd, ON, Canada) was used as secondary antibody. The signal was revealed with the streptavidin-biotin peroxidase reaction method using an ABC Vectastain elite kit (Vector Laboratories Inc) and 3-amino-9-ethylcarbazole (AEC, Sigma-Aldrich) as chromagen. Slides were counterstained with Mayer's hematoxylin.

## List of abbreviations used

ApoA-I: apolipoprotein A-I; apoA-II: apolipoprotein A-II; apoC-II: apolipoprotein C-II; apoH: apolipoprotein H; DIG: digoxigenin; GD: gestation day; LPL: lipoprotein lipase.

## Competing interests

The authors declare that they have no competing interests.

## Authors' contributions

MC carried out all laboratory manipulations and contributed to interpretation of the data. PRP and YT together conceived the project and analyzed the data. PRP supervised MC and wrote the manuscript. YT obtained funding and supervised the entire project. All the authors read and approved the final manuscript.
